# Callose deposited at soybean sieve element inhibits long-distance transport of *Soybean mosaic virus*

**DOI:** 10.1186/s13568-022-01402-0

**Published:** 2022-06-04

**Authors:** Jie Zhang, Na Liu, Aihua Yan, Tianjie Sun, Xizhe Sun, Guibin Yao, Dongqiang Xiao, Wenlong Li, Chunyan Hou, Chunyan Yang, Dongmei Wang

**Affiliations:** 1 State Key Laboratory of North China Crop Improvement and Regulation/ Hebei Key Laboratory of Plant Physiology and Molecular Pathology/College of Life Sciences, Hebei Agricultural University, Baoding, 071001 China; 2 Institute of Cereal and Oil Crops, Hebei Academy of Agriculture and Forestry Sciences, Shijiazhuang, 050035 China

**Keywords:** Callose, *Soybean mosaic virus*, Grafting, Long-distance transport

## Abstract

The function of callose and its deposition characteristics at phloem in the resistance to the long-distance transportation of *Soybean mosaic virus* (SMV) through phloem was studied. Two different methods of SMV inoculation were used in the study, one was direct friction of the virus on seedling leaves and the other was based on grafting scion and rootstock to create different resistance and sensitivity combinations. Veins, petioles of inoculated leaves and rootstock stems were stained with callose specific dye. Results from fluorescence microscope observation, pharmacological test, and PCR detection of SMV coat protein gene (SMV-*CP*) showed the role of callose in long-distance transportation of SMV through phloem during infection of soybean seedlings. When the inhibitor of callose synthesis 2-deoxy-D-glucose (2-DDG) was used, the accumulation of callose fluorescence could hardly be detected in the resistant rootstocks. These results indicate that callose deposition in phloem restricts the long-distance transport of SMV, and that the accumulation of callose in phloem is a main contributing factor for resistance to this virus in soybean.

## Introduction

*Soybean Mosaic Virus* (SMV) is the most common and severe disease for soybean [*Glycine max* (L.) Merr.] production. The disease does not only reduce yield but also seed quality. As a result, defense mechanisms to this disease have been intensively investigated using approaches of both plant physiology and plant pathology. It is now known that the success of infection depends on intracellular transport of the virus through plasmodesmata (PD) (Wang 2021, Rodriguez et al. 2014) and the rapid transport system through the phloem (Chang et al. 2016, Kappagantu et al. 2020). Our previous work showed that, in the incompatible combination between soybean varieties Jidou 7 and SMV strains N3, callose (or β-1,3-glucan) was found accumulating at 2 h post inoculation (hpi) and its amount reached maximum at 96 hpi (Li et al. 2012). However, this phenomenon did not occur in the compatible combination between soybean varieties Jidou 7 and SMV strains Sc-8. We also found that the fluorescence due to callose formation at the inoculation sites in the incompatible combination disappeared following the injection of the inhibitor for callose synthesis 2-DDG. At the same time, necrosis was observed in the incompatible combination and SMV was detected in the upper leaf which was not infected by virus. Results from immunohistochemical analysis showed that callose deposition at PD of infected sites is a key factor restricting the movement of the virus between cells during the defensive response of soybean to viral infection. Aphids are the main transmission medium of SMV in the field. They inject SMV into soybean phloem when sucking juice with piercing-sucking mouthparts. It is not clear what is the mechanism limiting the long-distance transport of virus in resistant genotypes.

Considerable knowledge on phloem structure and its role in molecular transport (Bendix and Lewis 2018) and virus movement has been accumulated (Hipper et al. 2013, Folimonova and Tilsner 2018). Results from recent studies indicate that phloem is highly responsive to virus infection, even more so than surrounding tissues (Collum et al. 2020). Due to the difficulty of studying phloem-specific processes, long-distance viral movement remains poorly characterized. Many aspects of viral movement are not fully elucidated and much of what is known only applies to specific systems (Bendix and Lewis 2018). Callose is a special component of cell wall or a substance binding to cell wall (Kumar et al. 2015, Schneider et al. 2016). Callose synthesis and decomposition directly relate to plant growth and development, playing an important role in vascular metabolism and gametophyte development (Sivaguru et al. 2000, Hao et al. 2008). Previous results also showed that callose deposit at sieve plate when planthopper was used to stimulate rice phloem cells, a result being treated as important resistant mechanism to resist planthopper injure (Hao et al. 2008). It has also been shown that cell wall-associated proteins and cadmium (Cd) ion-induced glycine-rich protein (cdiGRP) inhibit systemic phloem transport of vein-clearing virus (TVCV, genus Tobamo virus) in turnip when overexpressed (Ueki and Citovsky 2002). Interestingly, plants that over-accumulating cdiGRP display increased levels of callose in phloem and associated PD, providing a possible explanation for why viral movement is restricted in such plants (Iglesias and Meins 2000, Ueki and Citovsky 2002).

Our previous studies showed that the resistant levels of soybean genotypes to SMV were closely related to the speed of callose deposition at the sites of inoculation. Callose was detected sooner in genotypes with stronger resistance (Si Si et al*.* 2013). Recently, we found also that hydrogen peroxide (H_2_O_2_) signal was associated with the regulation of callose accumulation in restricting the cell-to-cell movement of SMV through plasmodesmata (Sun et al*.* 2021). The questions now are whether rapid accumulation of callose in the veins, petioles and stems can be detected from resistant genotypes following friction inoculation, and how phloem response when the virus is introduced directly into the tissue? To answer these questions, we grafted susceptible scion onto resistant root stock and then inoculated the scion thus allowing the virus to get into the phloem directly. Several methods were then used in the study reported here to investigate the roles of callose deposition in restricting long-distance transportation of the virus in soybean.

## Materials and methods

### Plant cultivation and virus inoculation

Four genotypes were used in this study. Three of them (including Jihuang 13, nf58 and Wuxing 2) were obtained from Grain and Oil Crops Research Institute, Hebei Academy of Agriculture and Forest Science. The other one, Nannong 1138–2, was obtained from the Soybean Institute, Nanjing Agricultural University. Both SMV strains used (SC-8 and N3) were both obtained from the Soybean Institute, Nanjing Agricultural University. SMV was propagated using the susceptible genotype Nannong 1138–2, and sap from infected leaves was used as virus inoculum based on the method described previously (Li et al. 2012). Our previous work has shown that Wuxing 2 had resistance level 0 to SMV strain N3 (Wuxing 2 and SMV strain N3 forms a incompatible combination), nf58 had resistance level 5 (nf58 and N3 forms a compatible combination) (Li et al. 2012). Seedlings were planted in an insect-proof greenhouse with a 14 h light/10 h dark cycle with light intensity of 700 μmol photons·m^−2^·s^−1^ and a constant temperature of 25 °C. Friction inoculation was carried out when the first trifoliate leaves were fully expanded (Li et al. 2012, Yao et al. 2010), and the inoculated leaves, petioles and stems were sampled at 24 hpi. Some samples were used for aniline blue staining and others were frozen in liquid nitrogen and then stored at − 80 °C refrigerator for RNA extraction. Simulated inoculations with water were used as controls.

### Grafting and sample preparation

Scion of nf58 or Jihuang 13 was grafted onto the rootstock Wuxing 2 based on the method described by Yao et al. (2010). SMV strain N3 was used to inoculate scion leaves of successfully grafted plants. The bud of the scion was grafted at the rootstock to induce the regeneration of the bud of the scion to form branches. Either Jihuang 13 or nf58 produces a compatible interaction with the virus while Wuxing 2 produces an incompatible interaction with this virus (Li et al. 2012, Yao et al. 2010). Three days after inoculation, the stem of Wuxing 2 were collected for callose staining and RNA extraction. Six days after inoculation, the infected nf58 leaves and the leaves from the rootstock were sampled for examining the disease symptoms (Si Si et al*.* 2013). As a scion donor, Jihuang 13 inoculated with SC-8, a compatible combination, was used as a positive control. SMV strain SC-8 with Jihuang 13 and Wuxing 2 were used as compatible combinations (Si Si et al*.* 2013).

### Fluorescent labeling of callose with aniline blue

Callose was stained with a modified method of the aniline blue fluorochrome procedure. Briefly, inoculated leaves were placed in a solution (containing 50% ethanol, 16.67% glycerol, 16.67% phenol, and 8.33% lactic acid, v/v) and boiled for 2 min, and then washed for 5 min with ddH_2_O (three times). The samples were then treated for 15 min in the staining solution (0.01% aniline blue dissolved in 0.1 mol L^−1^ PBS, pH 8.0). The treated leaves were then washed again with ddH_2_O and examined with a fluorescence microscope at Ex/Em = 385 nm/495 nm (Li et al. 2012, Conrath et al. 1998).

### 2-DDG treatment prior to SWV inoculation

Solid 2-DDG was dissolved in a solution of PBS (pH7.0), and 500 μmol L^−1^ 2-DDG were used to inject leaves using the method described previously (Si Si et al*.* 2013, Xiao et al. 2018). The 2-DDG treated plants were cultivated for 24 h before SMV inoculation.

### Detection of SMV-*CP* gene and its protein product

SMV-*CP* gene was identified as the SMV specific coat protein gene in a previously study (Li et al. 2012). The forward primer 5′-ATGCTCAGACAAGTGAGCT-3′ and reverse primer 5′-CTCCCTGCCATTCATAAAC-3′ for the gene were synthesized by Sangon Biotech company of Shanghai. The length of the PCR product from the gene using this primer pair was about 1050 bp.

Total RNA was isolated with an RNA extraction kit (UNlQ-10 Column Trizol Total RNA Isolation Kit, Sangon Biotech, Shanghai, China) and reverse-transcribed into cDNA with a reverse transcription kit (PrimeScript RT reagent Kit with gDNA Eraser, TaKaRa, Dalian, China). Jihuang 13 leaves inoculated with SC-8 were used as a positive control, and negative controls were non-infected leaves of either nf58 or Wuxing 2. RT-PCR assays were performed based on the method described by Li et al. (2012).

The expression of SMV CP protein product was detected using Western blotting. Rabbit polyclonal antibody prepared with recombinant SMV CP protein expressed in E. coli was used as primary antibody at a dilution ratio of 1:5000 and detected using HRP chemiluminescence. Detailed method of western blotting was done as previously described (Sun et al*.* 2021).

## Results

### Callose deposition in veins, petioles and stems following SMV inoculation

**Fig. 1:**
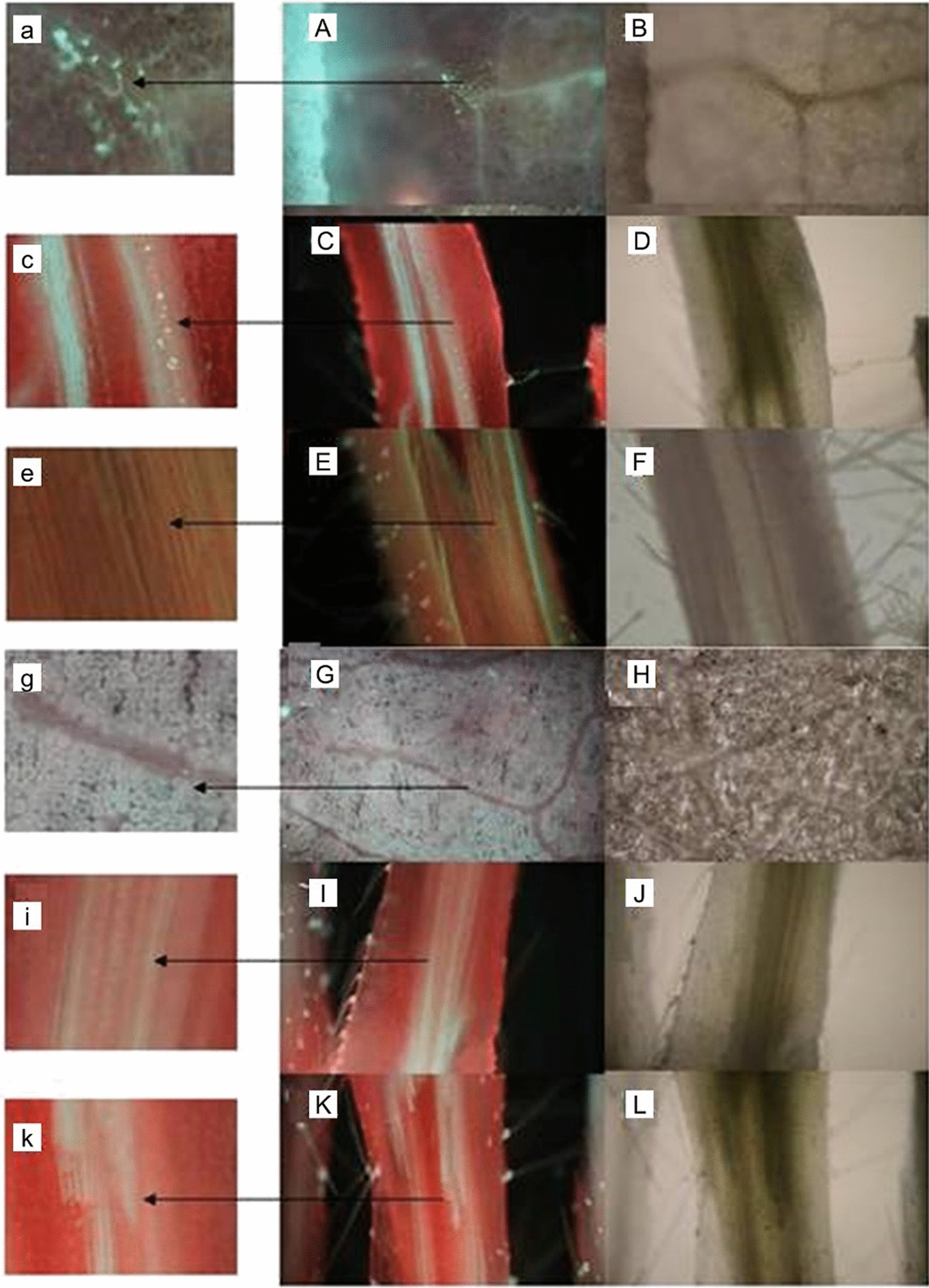
Callose in different tissues of plants following inoculation with SMV. **A** Fluorescence from stained callose in leaf vein of Wuxing 2 frictionally inoculated with N3; **B** micrograph of A under transmission light; **C** Fluorescence from stained callose in petiole of Wuxing 2 frictionally inoculated with N3; **D** micrograph of C under transmission light; **E** callose was not detectable in stem of Wuxing 2 frictionally inoculated with N3; **F** micrograph of E under transmission light; **G** callose was not detectable in vein of Wuxing 2 simulating inoculated; **H** micrograph of (**G**) under transmission light; **I** callose was not detectable in petioles of Wuxing 2 simulating inoculated; **J** micrograph of (**I**) under transmission light; K: callose was not detectable in stems of the Wuxing 2 inoculated with water; **L** micrograph of K under transmission light; a, c, e, g, i, k: enlarged pictures of the areas labelled with the arrows in picture (**A**, **C**, **E**, **G**, **I**, **K**)

SMV could directly enter phloem of leaf vein following friction inoculation. Punctate staining of callose was observed in the veins (Fig. 1A, B) and petioles (Fig. 1C, D) of resistant cultivar Wuxing 2 inoculated with N3 after 24 h. However, callose was not detectable in the stem of the plants at this point of time (Fig. 1E, F) nor from any of the tissues from plants of the compatible combinations (Jihuang 13 inoculated with SC-8, result not shown). As expected, callose was also not detected in veins, petioles or stems from the control genotype (Wuxing 2 inoculated with H_2_O) (Fig. 1G–L).

### SMV-*CP* gene detection in veins, petioles and stems of incompatible combinations

**Fig. 2:**
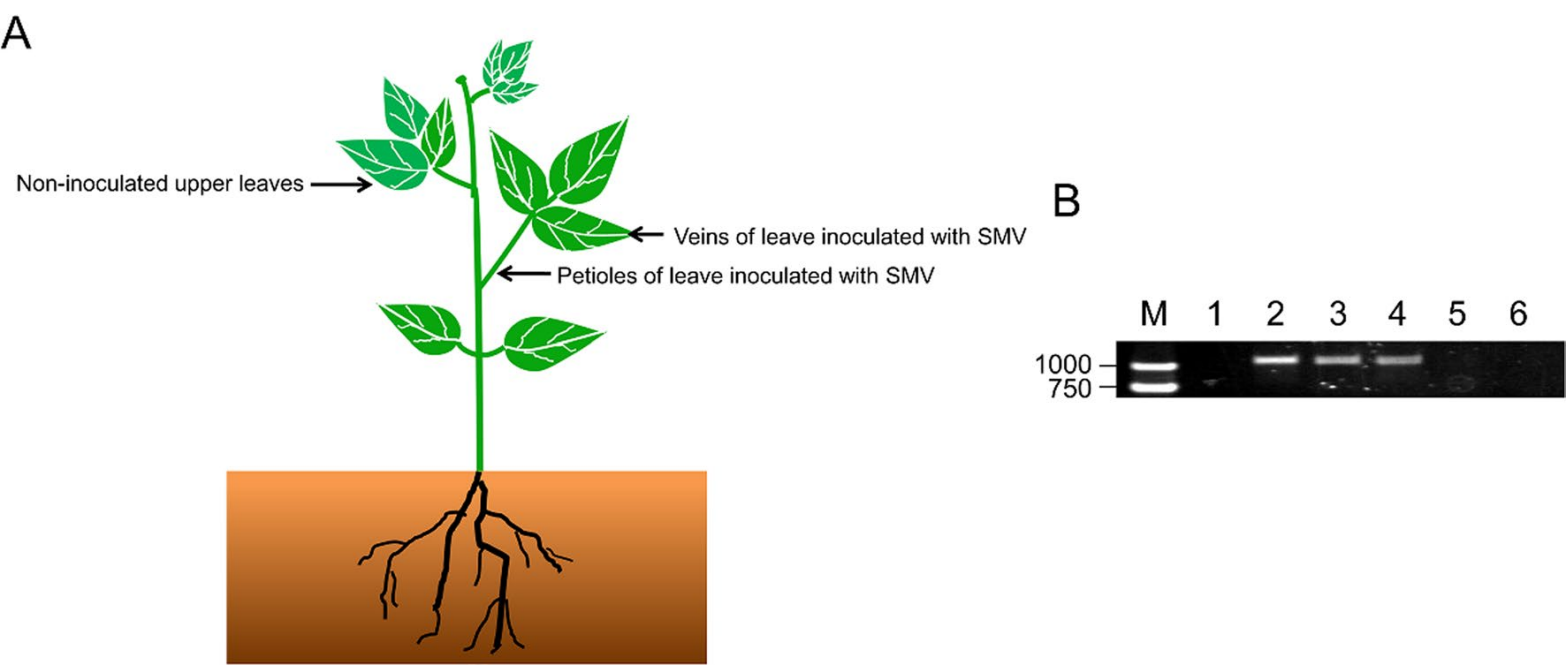
Spread of SMV in the incompatible host. **A** Schematic diagram showing plants inoculated with SMV; **B** Detection of SMV-*CP* gene by RT-PCR analysis from different tissues of soybean plants. M: DL2000bp DNA marker; 1: negative control (simulated inoculation with water); 2: positive control (Jihuang 13 leaves inoculated with SC-8); 3: the veins of inoculated leaves; 4: the petiole of inoculated leaves; 5: the stem; 6: non-inoculated upper leaves

To confirm the results observed with fluorescence staining, the SMV specific coat protein gene SMV-*CP* was analyzed. This gene was detected in the veins and petioles of the inoculated leaves from plants of the incompatible combination, but it was not detected in either the stem (schematic diagram in Fig. 2A) or non-inoculated upper leaves (Fig. 2A, B).

### Callose produced in the phloem of resistant cultivars hinders intercellular transport of SMV

**Fig. 3:**
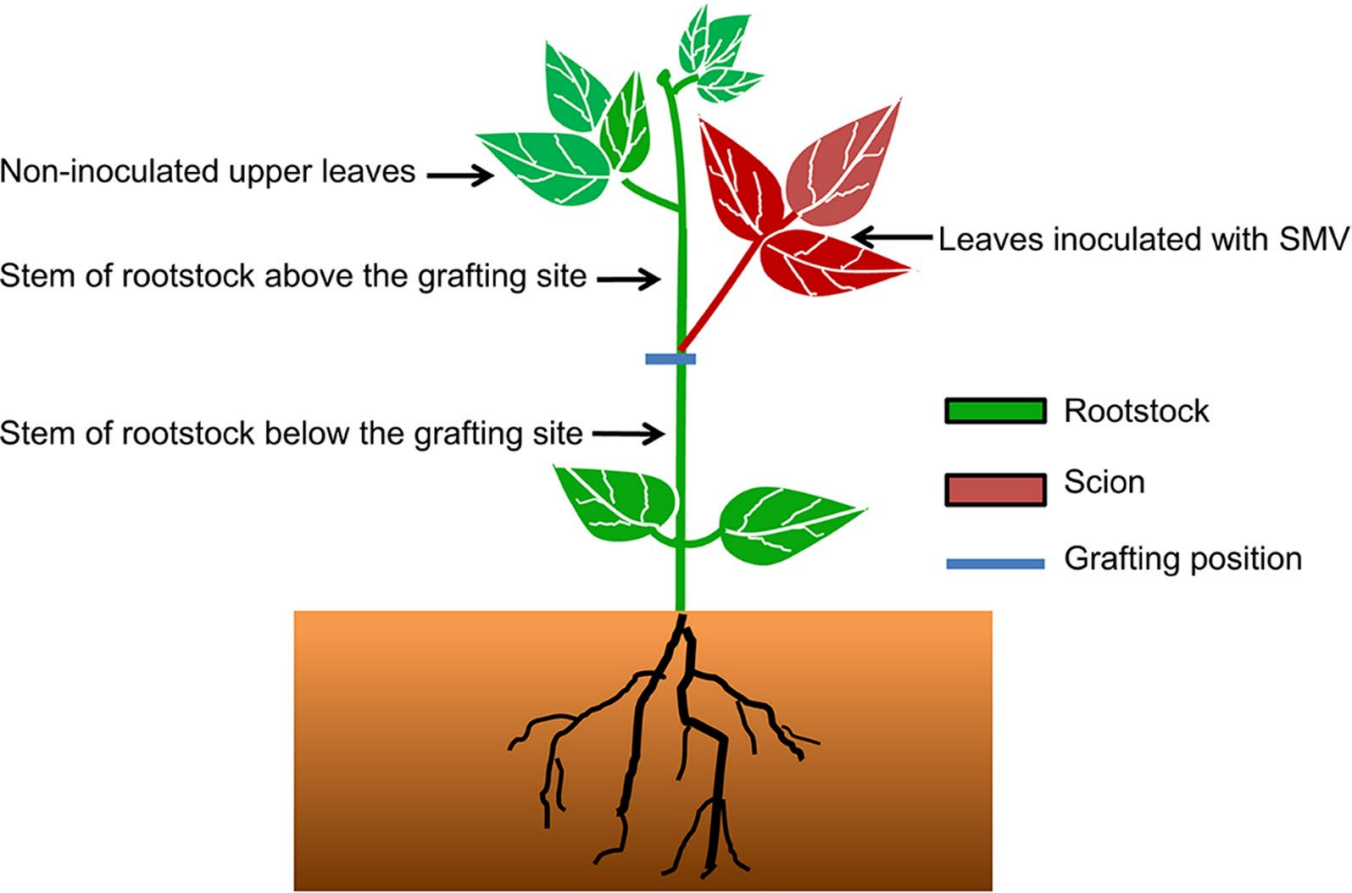
Schematic diagram showing virus inoculation of different resistant genotypes after grafting

**Fig. 4:**
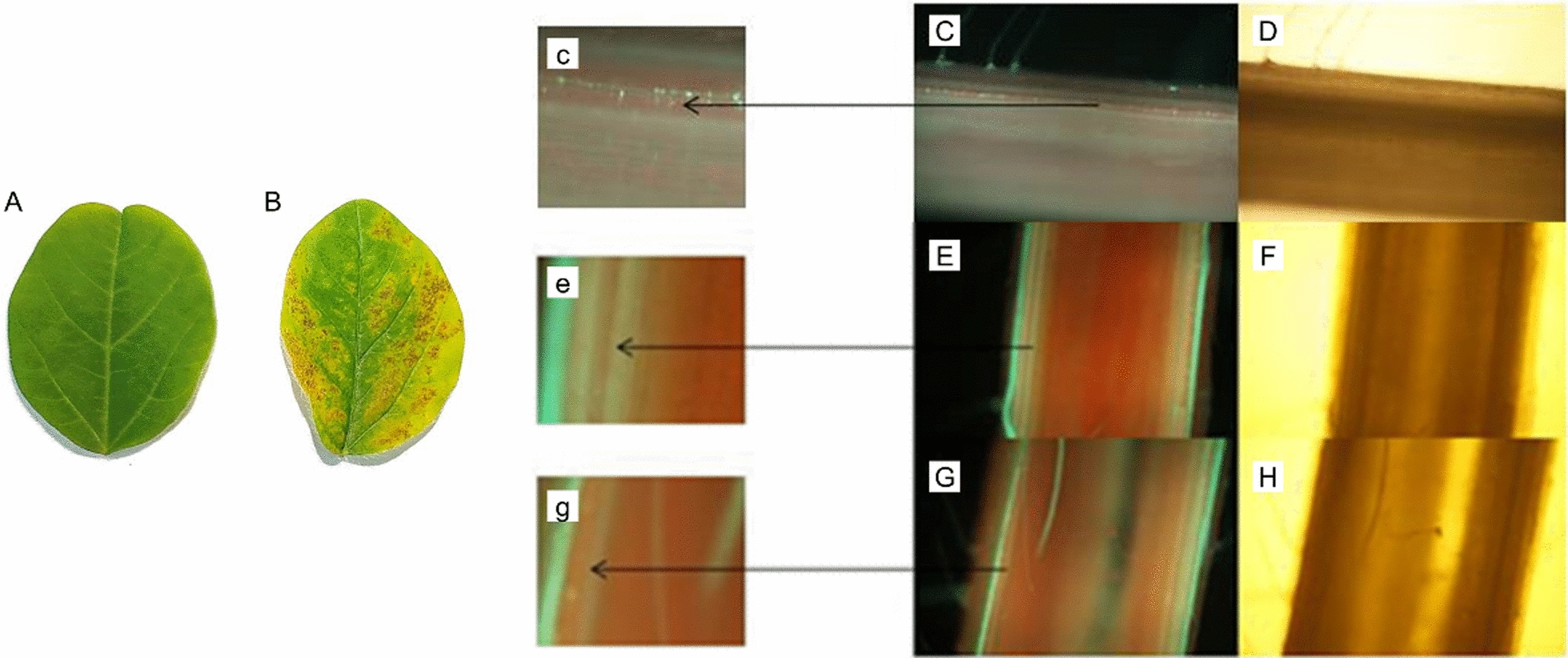
Callose in rootstock stem when scion was inoculated with SMV. **A** Non-inoculated upper leave of the rootstock; **B** Scion leaves inoculated with SMV; **C** Callose fluorescence was detected in stem of rootstock below the grafting site at 3-days after scion nf58 was frictionally inoculated with N3; **D** micrograph of C under transmission light; **E** Callose fluorescence was not detected in stem of rootstock above the grafting site at 3 days after scion nf58 was frictionally inoculated with N3; **F** micrograph of E under transmission light; **G** Callose fluorescence was not detected in stem base of the rootstock Wuxing 2 at 3-days after scion Jihuang 13 was frictionally inoculated with SC-8; **H** micrograph of (**G**) under transmission light; c, e, and g: the enlarged pictures of the areas labeled with arrows in picture C, E, and G

Due to the arbitrary method based on traditional friction inoculation, it is difficult to determine whether the virus would enter the phloem. To investigate the mechanism of long-distance transport of virus in the resistant genotypes, we conducted a grafting experiment (schematic diagram in Fig. 3). The susceptible cultivar was used as scion and grafted onto rootstock of resistant genotypes. Inoculating the scion of the grafted plants would allow the virus enter phloem of the rootstock directly. nf58 is used as scion and grafted onto 12 d old Wuxing 2 (at this point of time, the new leaves were fully expanded, and the stem was about 15–20 cm in length), and the scion was inoculated with the SMV strain N3 when the second leaves were full expanded. Six days later, necrotic spots appeared on inoculated leaves (Fig. 4A), but not on leaves of Wuxing 2 (Fig. 4B).

To assess callose accumulation, stems of rootstock Wuxing 2 were stained with aniline blue. Strong callose fluorescence was detected in the phloem of rootstock stem below the grafting site (Fig. 4C, D) but not in the phloem of rootstock stem above the grafting site (Fig. 4E, F). Callose was also not detected in the control where the scion of Jihuang 13 was inoculated with SC-8 (Fig. 4G, H). Necrotic spots appeared on the rootstock leaves 6 days after inoculation (not shown).

When scion nf58 was pre-treated with 2-DDG prior to SMV inoculation, weak fluorescence signal was detected in the stem of Wuxing 2 three days after SMV inoculation. Necrotic spots were observed in the upper leaves of the rootstock 6 days after inoculation (data not shown). These results indicate that, when the virus enters the phloem of resistant genotypes through grafting, callose induced by virus will deposit in the phloem, thereby limiting the long-distance transmission of the virus.

### SMV-*CP* gene analysis based on detection of callose deposition

**Fig.5:**
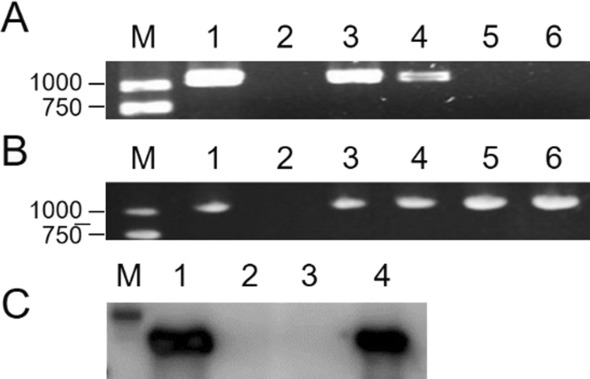
Detection of SMV-CP gene product. **A** Detection of SMV CP gene on grafted plants; M: DL 2000 bp DNA marker; 1: positive control (Jihuang 13 leaves inoculated with SC-8); 2: negative control (simulating inoculated leaves); 3: scion nf58 leaves after inoculation; 4: Stem of rootstock above the grafting site; 5: Stem of rootstock below the grafting site; 6: non-inoculated upper leaves of rootstock. **B** SMV-*CP* gene detection in rootstock when scion nf58 inoculated with N3 was pre-treated with 2-DDG. M:DL2000 bp DNA marker; 1: positive control (Jihuang 13 leaves inoculated with SC-8); 2: negative control (leaves inoculated with water); 3: upper leaves of Wuxing 2; 4: stem of rootstock above the grafting site; 5: stem of rootstock below the grafting site; 6: scion leaves after inoculation. **C** Western blotting detection of SMV CP. M: Easysee western marker; 1: Leaves of scion after inoculation with SMV; 2: Rootstock stem below the grafting site; 3: Uninoculated rootstock leaves above the grafting site; 4: Stem of rootstock above grafting site

SMV-*CP* gene based on RT-PCR was used to assess whether the virus is present in scion and rootstock. SMV-*CP* gene was detected in the leaves of scion nf58 and stem of rootstock Wuxing 2 below the grafting site when inoculated with SMV strain N3. However, the gene was not detected in the upper leaves nor the upper section of the rootstock stem (Fig. 5A). This result suggests that N3 could enter the phloem of Wuxing 2 through the veins of the scion nf58 which forms a compatible combination with N3. On the contrary, SMV entering the phloem of Wuxing 2 was restricted as this genotype and N3 form an incompatible combination.

SMV-*CP* gene was detected from inoculated leaves, upper and bottom sections of stem as well as the upper leaves of Wuxing 2 when 2-DDG was used to treat the nf58 scion prior to inoculation with N3 (Fig. 5B). The presence of SMV was also identified by Western blotting using an antibody that specifically recognizes the SMV CP protein product (Fig. 5C). These results indicate that introducing virus into the phloem by grafting induced callose accumulation in phloem, that callose accumulation in phloem of resistant genotypes limits the long-distance transport of virus, and that suppressing callose production by injecting 2-DDG enhances the long-distance transport of SMV strains in Wuxing 2.

## Discussion

Phloem is the principle route for viruses transportation, studying this tissue remains a challenge (Kappagantu et al. 2020). Infecting phloem can be achieved using phloem-feeding aphids, which is difficult to establish and requires specialized systems (Nelson et al. 2006). This traditional method of inoculation does not lead to successful infection of the phloem, which limits the research on the mechanism of long-distance virus transportation in the phloem of resistant genotypes.With the continuous improvement of the micro grafting technology (Marsch-Martínez et al. 2013, Sun et al. 2019), it has become an important method to study long-distance signal transmission in plants. The grafting technology system of soybean has been established in our team (Yao et al. 2010). Two soybean varieties Jihuang 13 and Jidou 7 were used as scions and rootstocks, respectively. SMV strain SC-8 was used to inoculate leaves of scion Jihuang 13 (Jihuang 13 and Jidou 7 were compatible with SMV strain SC-8). The SMV-*CP* gene of the virus was detected at 96 h after inoculation. It was found that the virus was transported from the leaves of scion Jihuang 13 to the stems of rootstock Jidou 7, indicating that grafting technology successfully solved the problem of virus inoculation in phleom, and providing technical support for the study of long-distance transportation of virus through phleom and the mechanism of long-distance transportation of the virus in resistant genotypes.

The obstacles facing the long-distance transportation include the plasmodesmata mediated selective symplastic transport between companion cells and bundle sheath cells (Peleg et al. 2007). During the process of loading and unloading, virus was restricted by different components, such as callose (Hao et al. 2008) and the N protein of enveloped plant virus (Zhang et al. 2012). Cowpea chlorotic spot virus (Goodrick et al*.* 1991) and tomato infertility virus (Thompson and García-Arenal 1998) were limited in plasmodesmata mediated selective symplastic transport between companion cells and bundle sheath cells. Callose usually deposited on the sieve plate of phloem (Lukan et al. 2018), Hao et al. found callose deposition in rice phloem stimulated using planthopper (Hao et al. 2008). *Sonchus yellow net virus* can replicate in phloem and spread through long-distance transportation. Complete virus particles or structural proteins exist in phloem, xylem parenchyma cells, and even xylem vessels and other components, but there is no direct evidence showing that complete virus particles can be spread through long-distance transportation via xylem vessels in plants (Zhou et al. 2019). Our results showed that callose accumulated and deposited in the veins of resistant genotype prevent virus long-distance transportation into stem from friction inoculated leaves. To clarify why virus cannot be transported freely in phloem, we pre-injected 2-DDG to the scions (susceptible genotypes) before inoculating with N3. Six days after virus inoculation, necrotic spots also appeared in the non-inoculated upper leaves of the rootstocks of the resistant genotype, and SMV-*CP* gene was also detected from them. The results indicated that callose accumulation was hindering the long-distance transportation of virus.

In what tissues does callose play the role in blocking the long-distance transport of the virus? It is well known that callose exists at the sieve plate of phloem (Kappagantu et al. 2020). To further explore that the blocking effect of callose on virus transport is caused by its deposition of sieve plate, we stained the stem of rootstock Wuxing 2 with the callose specific dye aniline blue. Preliminary laboratory work showed that the probable time of SMV delivered from the vaccination site to the phloem was 42 hpi, so the time callose was detected is three days after scion was inoculated with SMV. The results showed that more callose was accumulated in phloem of stem rootstock Wuxing 2 below the grafting site after scion nf58 was inoculated by N3, while weak fluorescence signal of callose was detected in the stem of Wuxing 2, and necrotic spots were observed in the upper leaves of the rootstock when scion was pre-treated with 2-DDG. These results indicated that callose depositing in the phloem of resistance genotypes is a major factor limiting the long-distance transport of the virus.

We have demonstrated in previous studies that callose plays an important role in restricting the cell-to-cell movement of SMV through plasmodesmata (Li et al. 2012, Sun et al*.* 2021). This study showed that lots of callose would accumulate in plant veins and petioles in the incompatible combination when infection was conducted by frictional inoculation and that would be important to host resistance to the long-distance transport of SMV. The virus was introduced into the phloem of resistant varieties by grafting technology, and it was found that the deposition of callose in the phloem was also the main factor limiting the long-distance transmission of the virus in soybeans. This study will help us deeply understanding of the molecular mechanism of soybean resistance to virus infection, enhance our understanding of the physiological mechanism of host resistance and susceptibility formation in the interaction between soybean and SMV. It also provides new ideas for anti-disease breeding and new strategies in controlling the virus, including genetic engineering.

## Data Availability

Not applicable.

## Abbreviations

SMV: *Soybean mosaic virus*
CP: Coat protein
2-DDG: 2-Deoxy-D-glucose
PD: Plasmodesmata
H_2_O_2_: Hydrogen peroxide

## References

[CR1] Bendix C, Lewis JD (2018). The enemy within: phloem-limited pathogens. Mol Plant Pathol.

[CR2] Chang CH, Hsu FC, Lee SC, Lo YS, Wang JD, Shaw J, Taliansky M, Chang BY, Hsu YH, Lin NS (2016). The nucleolar fibrillarin protein is required for helper virus-independent long-distance trafficking of a subviral satellite RNA in plants. Plant Cell.

[CR3] Collum TD, Stone AL, Sherman DJ, Rogers EE, Dardick C, Culver JN (2020). Translatome profiling of *Plum pox virus*-infected leaves in european plum reveals temporal and spatial coordination of defense responses in phloem tissues. Mol Plant Microbe Interact.

[CR4] Conrath U, Klessig DF, Bachmair A (1998). Tobacco plants perturbed in the ubiquitin-dependent protein degradation system accumulate callose, salicylic acid, and pathogenesis-related protein 1. Plant Cell Rep.

[CR5] Folimonova SY, Tilsner J (2018). Hitchhikers, highway tolls and roadworks: the interactions of plant viruses with the phloem. Curr Opin Plant Biol.

[CR6] Goodrick BJ, Kuhn CW, Hussey RS (1991). Movement of *Cowpea chlorotic mottle virus* in soybean with nonnecrotic resistance. Phytopathology.

[CR7] Hao P, Liu C, Wang Y, Chen R, Tang M, Bo Du, Zhu L, He G (2008). Herbivore-induced callose deposition on the sieve plates of rice: an important mechanism for host resistance. Plant Physiol.

[CR8] Hipper C, Brault V, Ziegler-Graff V, Revers F (2013). Viral and cellular factors involved in phloem transport of plant viruses. Front Plant Sci.

[CR9] Iglesias VA, Meins F (2000). Movement of plant viruses is delayed in a beta-1,3-glucanase-deficient mutant showing a reduced plasmodesmatal size exclusion limit and enhanced callose deposition. Plant J.

[CR10] Kappagantu M, Collum TD, Dardick C, Culver JN (2020). Viral hacks of the plant vasculature: the role of phloem alterations in systemic virus infection. Ann Rev Virol.

[CR11] Kumar D, Kumar R, Hyun TK, Kim JY (2015). Cell-to-cell movement of viruses via plasmodesmata. J Plant Res.

[CR12] Li W, Zhao YS, Liu CJ, Yao GB, Si Si Wu, Hou CY, Zhang MC, Wang DM (2012). Callose deposition at plasmodesmata is a critical factor in restricting the cell-to-cell movement of *Soybean mosaic virus*. Plant Cell Rep.

[CR13] Lukan T, Baebler Š, Pompe-Novak M, Guček K, Zagorščak M, Coll A, Gruden K (2018). Cell death is not sufficient for the restriction of *Potato virus Y* spread in hypersensitive response-conferred resistance in potato. Front Plant Sci.

[CR14] Marsch-Martínez N, Franken J, Gonzalez-Aguilera KL, de Folter S, Angenent G, Alvarez-Buylla ER (2013). An efficient flat-surface collar-free grafting method for *Arabidopsis thaliana* seedlings. Plant Methods.

[CR15] Nelson T, Tausta SL, Gandotra N, Liu T (2006). Laser microdissection of plant tissue: what you see is what you get. Annu Rev Plant Biol.

[CR16] Peleg G, Malter D, Wolf S (2007). Viral infection enables phloem loading of GFP and long-distance trafficking of the protein. Plant J.

[CR17] Rodriguez A, Angel CA, Lutz L, Leisner SM, Nelson RS, Schoelz JE (2014). Association of the P6 protein of *Cauliflower mosaic virus* with plasmodesmata and plasmodesmal proteins. Plant Physiol.

[CR18] Schneider R, Hanak T, Persson S, Voigt CA (2016). Cellulose and callose synthesis and organization in focus, what's new?. Curr Opin Plant Biol.

[CR19] Si Si Wu, Li WL, Xiao DQ, Hou CY, Wang DM (2013). Callose fluorescence labeling in the different soybean varieties resistant to *Soybean mosaic virus*. J Plant Genetic Resour.

[CR20] Sivaguru M, Fujiwara T, Šamaj J, Baluška F, Yang Z, Osawa H, Maeda T, Mori T, Volkmann D, Matsumoto H (2000). Aluminum-induced 1-3-beta-D-glucan inhibits cell-to-cell trafficking of molecules through plasmodesmata: a new mechanism of aluminum toxicity in plants. Plant Physiol.

[CR21] Sun Y, Huang D, Chen Xu (2019). Dynamic regulation of plasmodesmatal permeability and its application to horticultural research. Hortic Res.

[CR22] Sun T, Sun X, Li F, Ma N, Wang M, Chen Y, Liu N, Jin Y, Zhang J, Hou C, Yang C, Dongmei W (2021). H_2_O_2_ mediates transcriptome reprogramming during *Soybean mosaic virus*-induced callose deposition in soybean. Crop J.

[CR23] Thompson JR, García-Arenal F (1998). The bundle sheath-phloem interface of *Cucumis sativus* is a boundary to systemic infection by *Tomato Aspermy Virus*. Mol Plant Microbe Interact.

[CR24] Ueki S, Citovsky V (2002). The systemic movement of a tobamovirus is inhibited by a cadmium-ion-induced glycine-rich protein. Nat Cell Biol.

[CR25] Ueki S, Citovsky V (2005). Identification of an interactor of cadmium ion-induced glycine-rich protein involved in regulation of callose levels in plant vasculature. Proc Natl Acad Sci USA.

[CR26] Wang A (2021). Cell-to-cell movement of plant viruses via plasmodesmata: a current perspective on potyviruses. Curr Opin Virol.

[CR27] Xiao DQ, Duan XX, Zhang MS, Sun TJ, Sun XZ, Li FK, Liu Na, Zhang J, Hou CY, Wang DM (2018). Changes in nitric oxide levels and their relationship with callose deposition during the interaction between soybean and *Soybean mosaic virus*. Plant Biol.

[CR28] Yao GB, Li WL, Zhao YS, Zheng WY, Wang DM (2010). The application of graft technique in the soybean for investigation of *Soybean mosaic virus* transporting mechanism. J Agric Univ Hebei.

[CR29] Zhang Y, Zhang C, Li W (2012). The nucleocapsid protein of an enveloped plant virus, Tomato spotted wilt virus, facilitates long-distance movement of *Tobacco mosaic virus* hybrids. Virus Res.

[CR30] Zhou X, Sun K, Zhou X, Jackson AO, Li Z (2019). The matrix protein of a plant rhabdovirus mediates superinfection exclusion by inhibiting viral transcription. J Virol.

